# The Effects of Virtual Height Exposure on Postural Control and Psychophysiological Stress Are Moderated by Individual Height Intolerance

**DOI:** 10.3389/fnhum.2021.773091

**Published:** 2022-01-12

**Authors:** Diana Bzdúšková, Martin Marko, Zuzana Hirjaková, Jana Kimijanová, František Hlavačka, Igor Riečanský

**Affiliations:** ^1^Department of Behavioural Neuroscience, Institute of Normal and Pathological Physiology, Centre of Experimental Medicine, Slovak Academy of Sciences, Bratislava, Slovakia; ^2^Department of Applied Informatics, Faculty of Mathematics, Physics and Informatics, Comenius University in Bratislava, Bratislava, Slovakia; ^3^Social, Cognitive and Affective Neuroscience Unit, Department of Cognition, Emotion, and Methods in Psychology, Faculty of Psychology, University of Vienna, Vienna, Austria; ^4^Department of Psychiatry, Faculty of Medicine, Slovak Medical University in Bratislava, Bratislava, Slovakia

**Keywords:** virtual reality, fear of heights, stress, balance control, body sway, acrophobia, visual height intolerance, exposure therapy

## Abstract

Virtual reality (VR) enables individuals to be exposed to naturalistic environments in laboratory settings, offering new possibilities for research in human neuroscience and treatment of mental disorders. We used VR to study psychological, autonomic and postural reactions to heights in individuals with varying intensity of fear of heights. Study participants (*N* = 42) were immersed in a VR of an unprotected open-air elevator platform in an urban area, while standing on an unstable ground. Virtual elevation of the platform (up to 40 m above the ground level) elicited robust and reliable psychophysiological activation including increased distress, heart rate, and electrodermal activity, which was higher in individuals suffering from fear of heights. In these individuals, compared with individuals with low fear of heights, the VR height exposure resulted in higher velocity of postural movements as well as decreased low-frequency (<0.5 Hz) and increased high-frequency (>1 Hz) body sway oscillations. This indicates that individuals with strong fear of heights react to heights with maladaptive rigidity of posture due to increased weight of visual input for balance control, while the visual information is less reliable at heights. Our findings show that exposure to height in a naturalistic VR environment elicits a complex reaction involving correlated changes of the emotional state, autonomic activity, and postural balance, which are exaggerated in individuals with fear of heights.

## Introduction

Correct postural control is essential for our daily living and requires accurate integration of visual, proprioceptive, and vestibular sensory information (Horak, 2006; Assländer and Peterka, 2014). The sensory systems operate in a complex, interactive feedback loop that can be significantly affected by emotions (Salassa and Zapala, 2009). The balance mechanisms are able to compensate for a loss of information from one or two of these sensory systems and failure to make timely and adequate adjustments in the balance control makes a person vulnerable to falls in a changing environment (Honeine and Schieppati, 2014). To maintain proper balance and produce corresponding postural commands is especially challenging for people with fear of heights. About 30% of the adult population suffers from visual height intolerance, which reduces quality of life, causes behavioural constraints and avoidance of exposure to heights (Huppert et al., 2020). When exposed to heights, these individuals typically show anxiety, vertigo, unsteadiness, postural imbalance and gait insecurity, inner agitation, rapid heartbeat, sweating, drowsiness, and tremor (Huppert et al., 2020). The most common trigger situations of these symptoms are looking down from towers, hiking and mountaineering, climbing ladders, walking over a bridge, and looking down from a high-rise window (Brandt et al., 2015; Huppert et al., 2020). Height intolerance is thought to originate from an interaction between the psychological factors, mainly anxiety, and the physiological factors, such as a discrepancy between the visual, vestibular and somatosensory information used for the postural control (Teggi et al., 2019).

In the last few years, virtual reality (VR) has been increasingly used in a number of different contexts and populations, e.g., research on balance and motor rehabilitation, training of patients with postural instability or vestibular disorders (Chiarovano et al., 2017; Lei et al., 2019), assessment of fear of heights and anxiety (Meehan et al., 2005; Cleworth et al., 2012; Widdowson et al., 2016; Peterson et al., 2018), or therapy of several psychiatric disorders (Geraets et al., 2021). The advantage of VR is that it can provide a robust and naturalistic sensory experience in controlled, complex, and easily repeatable environments. Moreover, it allows placing subjects in a variety of environments that they may otherwise avoid due to fear or safety restrictions. Simultaneously, it provides a persuasive sense of presence where the users feel that they are in a real environment. As a result, fear-related changes in postural control can be studied using VR under a wide range of threatening situations within the safe confines of a lab or clinic. In the assessment of balance, VR can be used to present specific visual stimuli that challenge postural control (e.g., a motion of the visual scene) to evaluate postural reactions in healthy people and in patients with various sensory and balance problems (Chiarovano et al., 2015).

Although VR does not completely replace the natural environment, it may be useful in the treatment of specific phobias, including fear of heights. Several recent studies have shown that exposure to virtual height is in many aspects comparable to real-world height exposure (e.g., Cleworth et al., 2012; Robert et al., 2016; Chiarovano et al., 2017; Peterson et al., 2018). Healthy adults experience a significant deterioration in balance control while standing or walking on a VR-simulated elevated platform or unsafe ground (Simeonov et al., 2005; Brown et al., 2006; Davis et al., 2009; Cleworth et al., 2012, 2016; Peterson et al., 2018). For the assessment of postural stability in stressful (real or simulated) environments, it is important to take into account the concurrently experienced fear and anxiety that can significantly contribute to the resulting postural instability. Importantly, growing evidence indicates that both real and virtual height exposure evoke substantial psychological effects, such as decreased balance confidence, increased anxiety and arousal, as well as changes in posture (Simeonov et al., 2005; Davis et al., 2009; Huffman et al., 2009; Cleworth et al., 2012, 2016; Zaback et al., 2015, 2019, 2021). It has been shown that height represents a natural threat and thus a stressor which evokes the activation of the sympathetic autonomic nervous system (ANS), as indicated by increased electrodermal, cardiovascular, and/or neuroendocrine markers (Meehan et al., 2005; de Quervain et al., 2011; Diemer et al., 2016; Martens et al., 2019).

Despite the important knowledge provided by previous studies exploiting VR exposure to heights some significant gaps remain and await to be clarified. First, considering the psychological effects of virtual height exposure and their relationship to individual differences in visual height intolerance, the results are rather inconsistent regarding the stress-related activation of the ANS. For instance, it has been reported that exposure to height in VR increased heart rate (HR; Diemer et al., 2016), had no effect on HR (Raffegeau et al., 2020), or decreased HR (Simeonov et al., 2005), increased electrodermal activity (EDA; Simeonov et al., 2005; Cleworth et al., 2012; Diemer et al., 2016), or did not affect EDA (Peterson et al., 2018; Vermehren and Carpenter, 2020), casting doubts on the efficacy of VR simulation to induce a significant sympathetic arousal. In particular, Diemer et al. (2016) reported that virtual height exposure elevated HR and EDA also in subjects who did not suffer from fear of heights, whereas in the study by Wuehr et al. (2019) HR or EDA were not elevated, even in participants who reported high levels of visual height intolerance. Second, also inconsistent are the reports on the effect of virtual height exposure on postural balance. While Cleworth et al. (2012) found that body sway magnitude decreased during exposure to height in VR, several other studies reported the opposite effect, i.e., an increase in the magnitude of body sway (Wuehr et al., 2019; Raffegeau et al., 2020; Chander et al., 2021). Furthermore, it has been reported that postural reactions to simulated height are independent of individual height intolerance and the elicited fear (Wuehr et al., 2019), in contrast to real height exposure when postural adjustments were related to individual fear and anxiety levels (Davis et al., 2009). Apparently, variability in methods and variables (both independent and dependent including the type of VR environment, simulated altitude, selected parameters to measure psychological and physiological functions), but also methodological rigour (including sample size) could heavily contribute to the diversity of research outcomes across the previous reports. Crucially, none of the earlier studies adopted a complex approach to investigate the mutual relationship of the effects of virtual height exposure on psychological, autonomic and postural measures with respect to trait fear of heights. Importantly, such a complex research approach is necessary given that multiple factors are considered to play a causal role in fear of heights (Huppert et al., 2020).

In the present study, we thus investigated the effect of simulated exposure to height on the postural control and the psychological as well as the physiological markers of stress response in individuals with varying sensitivity to the stressor. For this purpose, the individuals were assessed for their fear of heights and divided into two equally large groups (*low* versus *high* fear). Since exposure to height is a natural threat, we expected both groups to show an increase in psychophysiological arousal as well as changes in body sway magnitude and velocity indicative of increased body stiffening (Adkin et al., 2000; Carpenter et al., 2001; Davis et al., 2009; Huffman et al., 2009; Cleworth et al., 2012). Postural stiffening is a protective motor reaction that prevents destabilisation from perturbations and is characterised by lower amplitude but higher frequency of body sway, especially in the direction of postural threat (Carpenter et al., 2001; Raffegeau et al., 2020). However, due to higher sensitivity of individuals with high levels of fear, we expected more intense psychophysiological response and impaired balance control in the high compared with the low fear group. Furthermore, we adopted robust measures of association to analyse the relationship of the psychological and physiological variables in order to better understand the complexity and the nature of the effects induced by virtual height exposure.

## Materials and Methods

### Participants

Forty-six individuals participated in the study, of which 42 (mean age 27.0 ± 6.1 years) completed the whole protocol (descriptive sample statistics is reported in Table 1). Four participants were excluded from further analysis – three volunteers were not able to complete the whole VR procedure and in one participant we obtained incomplete data due to technical issues. Inclusion criteria for participation were age between 18 and 35 years and recruitment on a volunteer basis. Exclusion criteria were vestibular and balance problems or other neurological and orthopaedic disorders that affect postural control. Excluded were also participants suffering from mental disorders other than acrophobia (however, no participant reported a history of acrophobia as a clinical diagnosis confirmed by a psychiatrist). Each participant had normal or corrected-to-normal vision. The participants were not repeated or extensive users of VR. The study was approved by the Local Ethics Committee and all participants signed informed consent in agreement with the Declaration of Helsinki before the start of the measurement.

**TABLE 1:** Sample characteristics.

	Mean ± SD or *N*
Age (years)	27.0 ± 6.1
Gender (male/female)	14/28
Height (cm)	171.8 ± 7.6
Weight (kg)	66.2 ± 12.3
BMI (kg/m^2^)	22.3 ± 3.1
Trait anxiety (STAI-T score)	39.7 ± 8.1

### Design and General Procedure

The study had a mixed factorial experimental design (see section “Statistical Analysis” for more details). The measurements were carried out within a single session. Each participant first completed a short introductory interview aimed at collecting basic demographic data and explaining upcoming procedures. Then, the experimental procedure started, including five successive stages in the order: *baseline*, *virtual height (VH) 1–3*, and *recovery* (for more detail see Supplementary Figure 1). Self-reported levels of affective state [perceived distress (PD), see section “Self-Reported Psychological Measures”] and physiological data (see section “Measures of Autonomic Nervous System Activity and Signal Processing”) were assessed at all five stages; postural data were collected in *VH* stages. During the *baseline* and the *recovery* stages, the participants were comfortably seated and left alone in a quiet room for approximately 10 min while being immersed in a relaxing virtual environment. During *VH* stages, the participants were standing upright in the middle of an in-built force plate and were exposed subsequently (without interrupting the immersion to VR environment) to three virtual heights: 0 m (*VH1*), 20 m (*VH2*), and 40 m (*VH3*) in fixed order (also see section “Virtual Reality”). Physiological data were registered continuously throughout the *VH* stages. At each height, the participants were first asked to look around to perceive the simulated environment and then the level of psychological distress was assessed using a questionnaire. This required about 3 min, depending mainly on individual response time. Subsequently, postural measurements were carried out for 50 s. In *VH1* and *VH3* (i.e., at the ground level and the highest elevation), after obtaining (static) postural measurements, a response to a vibratory stimulation of lower leg muscles was assessed (stimulation duration = 7 s, measurement epoch duration = 20 s). Results of these dynamic epochs are not reported here. For safety reasons, one experimenter was standing close to the participant throughout the experiment, for the case the participant would show postural instability or a tendency to fall.

### Virtual Reality

**FIGURE 1:**
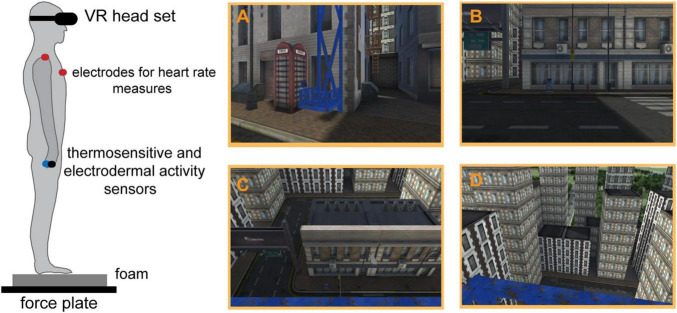
The schematic depiction of the experimental set up (left) and the main VR simulation (right). Panels **(A–D)** depict the virtual elevator and the participant’s view at the height of 0, 20, and 40 m, respectively.

Virtual reality simulations were performed using Oculus Rift (Facebook Inc., CA, United States), featuring two OLED screens (one per eye) running at 90 Hz with resolution 1080 × 1200 pixels and approximately 100° field of view (the interpupillary distance was adjusted for each participant). Two infrared sensors (Oculus Sensors) were used for 360° positional tracking (6 degrees of freedom). The sensors were positioned approximately 2 m apart from each other and 1.5 m apart from the participant’s position (i.e., the in-built force plate). Before the experimental session, the virtual area was calibrated at 2 m × 2 m and re-calibrated when necessary. Two distinct VR simulations were included within each session. The first was a relaxing virtual environment (Guided Meditation VR software), which was run at the *baseline* and *recovery* stages. The relaxing environment was used to familiarise participants with VR, provide a condition for the assessment of baseline psychological and physiological parameters, and recover the participants from the stressful situation of height exposure. The second was the virtual elevator with ∼1 m^2^ surface area in an urban environment, run during the stages *VH1–VH3* (see Figures 1A–D). Here, the participants were positioned at the centre of the virtual elevator with their toes aligned 10 cm away from the front edge of the platform without the possibility to take a step forward for compensation. The elevator was operated/moved by one of the experimenters to achieve the height of 20 m and 40 m (each transition lasted approximately 60 s). After the last stage at 40 m (*VH3*), the elevator descended back to 0 m and the urban simulation was switched back to the relaxing simulation (*recovery* stage), during which the participants were seated again. The participants’ experience in the virtual environment was evaluated at the end of the session using a short questionnaire adapted from previous works (Witmer and Singer, 1998; Schubert et al., 2001) assessing the technical quality of the simulation (comfort, smoothness, graphics, movement) and the VR experience itself (the level of immersion, authenticity, and real-life emotions; see Supplementary Material). Overall, the participants indicated that the technical quality as well as the experience was high [i.e., median = 3 (high) in the range from 0 (very low) to 4 (very high), for both measures].

### Self-Reported Psychological Measures

*Perceived distress* was assessed, using a procedure customised to be suitable for the application during the VR simulations. While exposed to the VR simulation, participants verbally reported their perceived level of distress using 11 items with a 6-point Likert scale ranging from 0 (no distress) to 5 (extreme distress). Based on our previous studies (Marko and Riečanský, 2018; Buzgoova et al., 2020), the items were selected to indicate psychological distress (“*agitation*,” “*tension*,” “*fear*,” “*panic*”), stress-related bodily sensations (“*heartbeat*,” “*tight stomach*,” “*muscular tension/stiffness*,” “*sweating*”), and cognitive appraisal of the simulation (“*worries*,” “*uncertainty*,” “*preoccupations*”). The item scores were summed to yield a total score indicating PD for each stage of the procedure. Cronbach’s α > 0.8 indicated high internal consistencies of the PD scale at all stages of the procedure. Finally, for each participant, the change in PD (ΔPD) was calculated as the difference between the average PD during the main simulation (stages *VH1–VH3*) and PD at the baseline stage. The ratings at zero height (stage *VH1*) were included in the average score to account for the individual differences in anticipatory distress.

**FIGURE 2:**
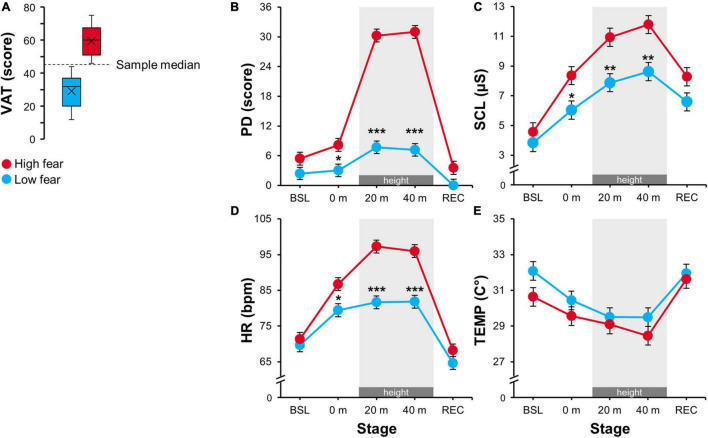
**(A)** Visual acrophobia/anxiety test (VAT) score distribution for the *low* (VAT < 45) and the *high* (VAT > 45) fear group. **(B–E)** Estimated marginal means for the physiological and psychological parameters: PD, perceived distress; SCL, skin conductance level; HR, heart rate; TEMP, peripheral skin temperature in 0, 20, and 40 m virtual heights and also in baseline (BSL) and recovery (REC) stages. Error bars represent ± SEM. Grey areas highlight exposure to virtual height (20 and 40 m). Significant differences between the groups are marked as follows: *p < 0.05, **p < 0.01, ***p < 0.001.

Furthermore, trait fear of heights and trait anxiety were assessed at the end of the session. *Fear of heights* was evaluated using a specific visual acrophobia/anxiety test (VAT) developed for the purpose of this study. VAT included a set of 11 randomly presented pictures (items) showing various situations involving heights. These items, representing a set of triggers, are akin to those used in previous studies (Cohen, 1977; Huppert et al., 2017). For each item, participants were instructed to examine the picture including a short descriptive caption and then indicate how anxious they would have felt in the depicted situation, using a scale ranging from 1 (no anxiety) to 7 (extreme anxiety). The item scores were summed to indicate the individual levels of fear of heights. Cronbach’s α > 0.95 indicated a very high internal consistency of the VAT scores. Notably, the VAT score strongly correlated (Pearson’s *r* = 0.84, *p* < 0.001) with a validation scale based on standard diagnostic criteria for acrophobia according to the Diagnostic and Statistical Manual of Mental Disorders (DSM, 2013). The validation scale included 6 statements (e.g., “*I get immediately anxious when being in height*”), for which individuals indicated a degree of agreement, using an ordinal response scale ranging from 0 (not at all) to 3 (definitely yes). The median of the VAT scores was used as the cutoff threshold, based on which the sample was divided into two groups of 21 individuals representing *low* (VAT < 45) and *high* (VAT > 45) level of fear of heights, respectively (see Figure 2A). *Trait anxiety* was assessed using the State and Trait Anxiety Inventory (STAI; Müllner et al., 1980; Spielberger et al., 1983) as both a descriptive and a control measure. Notably, VAT was not significantly correlated with STAI-T (Pearson’s *r* = 0.06, *p* = 0.707), confirming that VAT represents a specific measure targetting the distress and anxiety related to heights.

### Measures of Autonomic Nervous System Activity and Signal Processing

Physiological data, consisting of three distinct measures of sympathetic activity, were acquired using Neurobit Optima 4 (Neurobit Systems, Gdansk, Poland) and processed in LabChart 7 (ADInstruments, Otago, New Zealand).

*Skin conductance level (SCL)* was measured by a pair of reusable Ag/AgCl electrodes attached to medial phalanxes of the index and ring fingers (palmar side) of the participant’s non-dominant hand. SCL was derived from the electrodermal signal sampled at 15.625 Hz, which was digitally filtered (0.05 Hz low-pass filter) and smoothed (triangular 30-s moving window) to eliminate the phasic component of the EDA (i.e., skin conductance responses).

*Heart rate* was measured using two disposable 80 mm Ag/AgCl electrodes placed over the right clavicula and the left hypochondrium (an approximate of Einthoven’s lead II). HR was derived from R–R intervals of digitally filtered (5–35 Hz band-pass filter) electrocardiographic signals, recorded at a sampling rate of 1000 Hz. The HR signal was digitally filtered (0.05 Hz low-pass filter) and smoothed (triangular 30-s moving window) to exclude high-frequency and respiratory artefacts.

*Peripheral skin temperature (TEMP)* was measured using a thermosensitive sensor attached to the dorsal medial phalanx of the middle finger of the non-dominant hand and recorded at a sample rate of 15.625 Hz.

Finally, separately for each individual and physiological measure, average levels of sympathetic activity were extracted from the processed signals using 2-min segments (sampling windows) at each stage of the experiment (i.e., *baseline*, *VH1–VH3*, and *recovery*). Furthermore, we calculated the change in the level of sympathetic activation (i.e., ΔSCL, ΔHR, and ΔTEMP) as the difference between the average level of the respective measure during the main simulation (stages *VH1–VH3*) and the baseline level.

### Postural Measures and Signal Processing

Participants were standing relaxed on the in-built force plate, barefoot, with their feet parallel and at hip width, arms along the body and head in straight position (checked by the experimenter before starting each trial). During the measurements, the participants were asked to fixate a selected object placed at a distance of 5 m at eye level within the urban VR environment (the same for all participants) to ensure a standard visual input from the simulated scene. The duration of each epoch of postural data acquisition was 50 s. The postural responses were quantified by the displacement of the centre of foot pressure (CoP) measured by a custom-made force plate (45 cm × 45 cm × 6.5 cm), equipped with an automatic weight correction (for more details, see Hirjaková et al., 2017). Prior to the trials of the stage *VH1*, data were collected at virtual height 0 m while participants were standing on a firm support. All subsequent measurements (*VH1–VH3*) were carried out using a foam pad (50 cm × 41 cm × 6 cm, Airex Balance Pad, Switzerland) located on the force plate, which was used to amplify the destabilising effect of height and modify the proprioceptive information from feet (Jeka et al., 2004; Simeonov et al., 2005; Chiarovano et al., 2015). To obtain identical postural configuration between the trials, marks of exact position of the feet were placed on the force plate/foam. The CoP displacement in anterior–posterior (AP) and medio-lateral (ML) directions was recorded at a sample rate of 100 Hz and processed with a second-order low-pass Butterworth filter with a cutoff frequency of 5 Hz to eliminate low-amplitude measurement noise. Data were analysed and evaluated with MATLAB^®^ software (MathWorks, Inc., Natick, MA, United States). The bias from the CoP signal was removed to provide correct calculations of postural measures. Four postural parameters were calculated: *the root mean square* of the CoP displacement in AP *(RMS_*AP*_)* and ML *(RMS_*ML*_)* directions and *the mean velocity* of the CoP displacement in AP *(V_*AP*_)* and ML *(V_*ML*_)* directions. Based on previously published results demonstrating greater effect of height-induced threat in the direction of facing the threat (Adkin and Carpenter, 2018; Zaback et al., 2019, 2021), the CoP displacement in AP direction was subjected to spectral analysis, following which *the mean power frequency* (MPF) and *the power spectrum density* (PSD) were obtained in all *VH* stages (0, 20, and 40 m). The power spectrum was calculated from 0 to 3 Hz by fast Fourier transformation and was then divided into six frequency bands of interest as follows: the low-frequency band (0–0.5 Hz), the medium-frequency band (0.5–1 Hz), and the high-frequency bands (1–1.5, 1.5–2, 2–2.5, and 2.5–3 Hz). The bands selection was done based on our previous work (Hirjaková et al., 2017) and the research associating the low frequencies with contribution of visual information to body sway, medium frequencies to vestibular and somatosensory, and high frequencies to proprioceptive information (Golomer et al., 1994; Nagy et al., 2004). Similar frequency bands were recently used, e.g., by Johnson et al. (2019) and Zaback et al. (2019, 2021) to explore the threat-related postural adaptations. For each postural measure, a change score (i.e., ΔV_*AP*_, ΔRMS_*AP*_, etc.) was calculated as the difference between the average level of the respective measure at heights (i.e., 20 and 40 m) and at the ground level (i.e., 0 m).

### Statistical Analysis

The data were processed in JASP (JASP Team, version 0.14, 2020) and R studio (RStudio Team, 2019), using R language (R Core Team, 2019). Prior to statistical analyses, the data were screened for distributional properties and the measures showing outlying observations (i.e., values exceeding median ±1.5 interquartile range) were winsorised using two-sided 20% trimming (separately for each stage and group). Thereafter, the main hypotheses were evaluated using linear mixed effect models [LMEMs; lme4 package (Bates et al., 2015)] that included a fixed within-subject effect *block* (five levels: baseline, *VH1–VH3*, recovery), a fixed between-subject effect *group* (low versus high fear), their interaction, and a random intercept effect for each participant (default unstructured covariance matrix). Note that the postural measures were assessed only in the *VH* stages, thus the within-subject factor *block* had three levels for postural analyses. All LMEMs were fitted using restricted maximum likelihood (REML) and *p*-values were derived with Satterthwaite approximation for degrees of freedom, as these were shown to produce optimal estimates even for smaller samples (Luke, 2017). The *p*-values for pair-wise contrasts were corrected with Holm adjustment to account for family-wise error rate (adjusted *p*-values are reported). The semi-partial *R*^2^ was computed to estimate effects sizes for the LMEM analyses. Finally, the change scores from psychophysiological and postural measures were assessed using robust percentage-bend correlation analysis (20% bending constant).

## Results

### Psychological and Autonomic Nervous System Activity Measures

The LMEM for PD revealed a significant main effect of *block* and *group* and their interaction (Table 2). As shown in Figure 2B, the high fear group reported significantly higher PD compared to the low fear group at all three simulated heights (*p*_holm_ ≤ 0.01), in particular at 20 and 40 m (*p*_holm_ < 0.001). Otherwise, both groups showed very low PD at the *baseline* and *recovery* stages, where the difference was not significant (*p*_holm_ ≥ 0.085).

**TABLE 2:** Experimental effects induced by exposure to virtual height: summary of linear mixed effect models for psychophysiological measures.

Measure	Effect	*df*	*F*	*p*	*R* ^2^
PD	Group	1, 40	103.163	** < 0.001**	0.721
	Block	4, 160	128.079	** < 0.001**	0.762
	Group × Block	4, 160	50.082	** < 0.001**	0.556
SCL	Group	1, 40	7.262	**0.010**	0.154
	Block	4, 159	190.032	** < 0.001**	0.827
	Group × Block	4, 159	8.981	** < 0.001**	0.184
HR	Group	1, 39	14.033	** < 0.001**	0.265
	Block	4, 155	272.962	** < 0.001**	0.876
	Group × Block	4, 155	23.425	** < 0.001**	0.377
TEMP	Group	1, 40	1.588	0.215	0.038
	Block	4, 159	40.627	** < 0.001**	0.505
	Group × Block	4, 159	1.427	0.228	0.035

*The models included the effect of block (within-subject factor: baseline, VH1–VH3, and recovery stage), group (between-subject factor: high versus low fear of heights), and their interaction. PD, perceived distress; SCL, skin conductance level; HR, heart rate; TEMP, peripheral skin temperature. Significant effects are bolded.*

Furthermore, the virtual height affected all selected parameters of ANS activity. For SCL (μS) and HR (bpm), the LMEMs revealed a significant main effect of *block* and *group* as well as their interaction (Table 2). As shown in Figures 2C,D, SCL and HR continuously increased with ascending virtual height in both groups. However, the high fear group showed higher sympathetic activation at all heights (*p*_holm_ ≤ 0.023), while these differences were more substantial at 20 and 40 m (*p*_holm_ ≤ 0.002). There were no significant differences in SCL or HR at the *baseline* and *recovery* stages between the groups (*p*_holm_ ≥ 0.100). Finally, for TEMP (°C), the LMEM showed only a significant effect of *block*, while the effect of *group* and their interaction were not significant (Table 2). As indicated in Figure 2E, both groups showed a decrease in TEMP across the virtual heights. Although the high fear group yielded lower TEMP than the low fear group, this was not significantly different at any stage (*p*_holm_ ≥ 0.246).

### Postural Measures

First, we compared standing on the foam versus firm support at ground level (height 0 m). The LMEMs showed a significant main effect of *surface* on all postural parameters as well as a significant interaction of *group* and *surface* on V_*ML*_ and RMS_*ML*_ (for more details, see Supplementary Table 1 and Supplementary Figure 2). The analyses confirmed that all participants, regardless of fear of heights, showed larger and faster body sway when standing on the foam, with significantly higher V_*ML*_ (*p*_holm_ = 0.037) and RMS_*ML*_ (*p*_holm_ = 0.026) in the low fear group.

**FIGURE 3:**
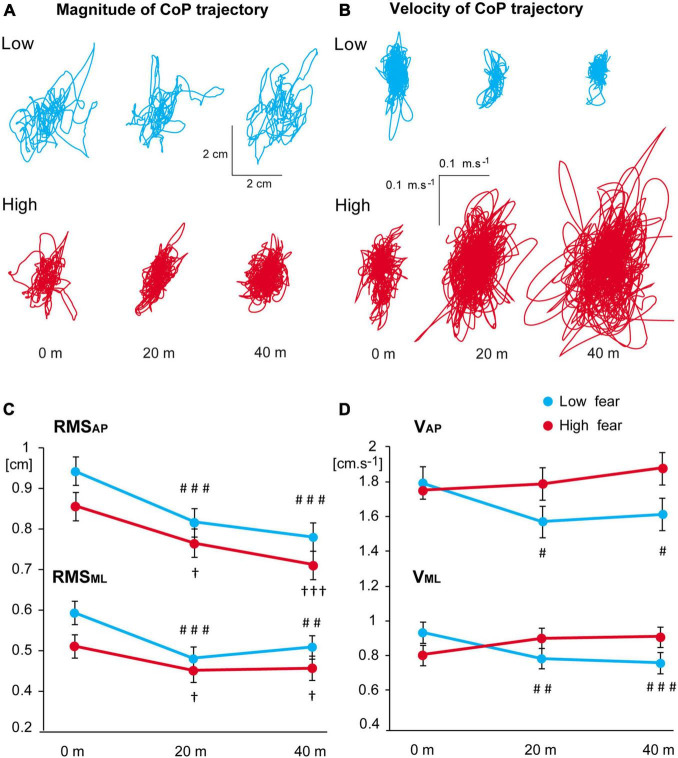
**(A,B)** Representative examples of individual trajectories in participants in the low and high fear groups during exposure to virtual height: **(A)** magnitude of the CoP displacement, **(B)** velocity of the CoP displacement. **(C,D)** Estimated marginal means ± SEM of the RMS **(C)** and velocity **(D)** of CoP deviations in both directions in 0, 20, and 40 m virtual heights in the low and high fear groups. Significant differences between heights 0 versus 20 m and 0 versus 40 m are marked as follows: ^#^*p* < 0.05, ^##^*p* < 0.01, ^###^*p* < 0.001 for the low fear group and ^†^*p* < 0.05, ^†††^*p* < 0.001 for the high fear group.

The virtual height exposure induced obvious alterations of the CoP displacement compared to virtual ground level in each participant (Figures 3A,B). The LMEMs revealed a significant effect of *block* on RMS_*AP*_ and RMS_*ML*_ (Table 3). RMS values decreased with ascending height comparably in both groups (Figure 3C). For V_*AP*_ and V_*ML*_, a significant interaction of *block* and *group* was found (Table 3). While the velocity of CoP displacement in both directions slightly decreased at virtual heights when compared with the ground level in the low fear group, in the high fear group the velocity increased with ascending virtual height (Figure 3D).

**TABLE 3:** Experimental effects induced by exposure to virtual height: summary of linear mixed effect models for postural measures.

Measure	Effect	*df*	*F*	*p*	*R* ^2^
V_*AP*_	Group	1, 40	1.656	0.206	0.040
	Block	2, 80	1.441	0.243	0.035
	Group × Block	2, 80	4.482	**0.014**	0.101
V_*ML*_	Group	1, 40	0.377	0.543	0.009
	Block	2, 80	0.712	0.494	0.017
	Group × Block	2, 80	11.843	** < 0.001**	0.228
RMS_*AP*_	Group	1, 40	2.570	0.117	0.060
	Block	2, 80	25.395	** < 0.001**	0.388
	Group × Block	2, 80	0.306	0.737	0.008
RMS_*ML*_	Group	1, 40	2.256	0.141	0.053
	Block	2, 80	13.528	** < 0.001**	0.253
	Group × Block	2, 80	1.131	0.328	0.027

*The models included the effect of block (within-subject factor: VH1–VH3), group (between-subject factor: high versus low fear of heights), and their interaction. V_AP_, velocity of CoP displacement in anterior–posterior direction; V_ML_, velocity of CoP displacement in medio-lateral direction; RMS_AP_, root mean square CoP deviation in anterior–posterior direction; RMS_ML_, root mean square CoP deviation in medio-lateral direction. Significant effects are bolded.*

**FIGURE 4:**
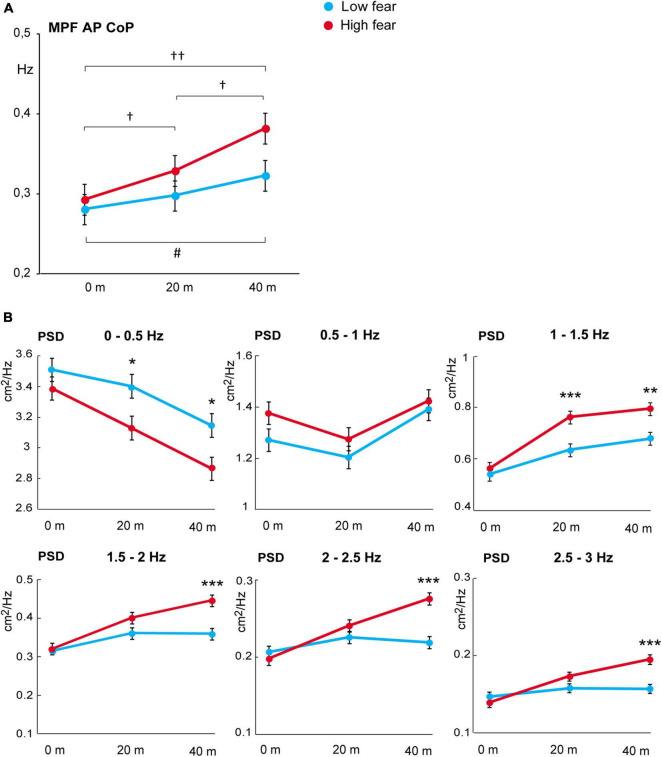
**(A)** Mean power frequency score of CoP displacement in anterior–posterior direction (estimated marginal means ± SEM). Significant differences between heights 0 versus 20 m, 0 versus 40 m, and 20 versus 40 m are marked as follows: ^#^*p* < 0.05 for the low fear group and ^†^*p* < 0.05, ^††^*p* < 0.01 for the high fear group. **(B)** Power spectral density of CoP displacement in anterior–posterior direction (estimated marginal means ± SEM) in six frequency bands at virtual heights of 0, 20, and 40 m in the low and high fear groups. Significant differences between groups are marked as follows: *p < 0.05, **p < 0.01, ***p < 0.001.

Next, we used frequency analysis to assess the effect of virtual height on postural body sway in more detail (also see Supplementary Figure 3). The LMEMs for MPF revealed a significant main effect of *block* [*F*_(2,80)_ = 10.278, *p* < 0.001, *R*^2^ = 0.204]. The effect for *group* [*F*_(1,40)_ = 2.829, *p* = 0.100, *R*^2^ = 0.066] and the interaction of *group* and *block* [*F*_(2,80)_ = 1.31, *p* = 0.275, *R*^2^ = 0.032] were not significant. The score of MPF did not significantly differ between the groups at any of the simulated heights (*p*_holm_ ≥ 0.076) (Figure 4A). The LMEMs for PSD indicated significant main effects of *block*, *group*, and their interaction at almost all analysed frequencies (Table 4). Further *post hoc* analyses revealed significant differences in the PSD mean values between low and high fear groups (Figure 4B). In the frequency range 0–0.5 Hz, body oscillations decreased with ascending virtual height in both groups, but the participants with high fear showed less oscillations at heights 20 and 40 m compared with low fear individuals (*p*_holm_ = 0.025, Figure 4B). On the other hand, oscillations in the frequencies above 1 Hz increased with ascending virtual height and were more pronounced in the individuals with high fear, in particular at the highest height.

**TABLE 4:** Experimental effects induced by exposure to virtual height: summary of linear mixed effect models for the PSD mean values of CoP displacement in anterior–posterior direction in selected frequency ranges.

Measure (Hz)	Effect	*df*	*F*	*p*	*R* ^2^
0–0.5	Group	1, 40	7.737	**0.008**	0.162
	Block	2, 80	26.827	** < 0.001**	0.401
	Group × Block	2, 80	1.082	0.344	0.026
0.5–1	Group	1, 40	2.444	0.126	0.058
	Block	2, 80	10.06	** < 0.001**	0.201
	Group × Block	2, 80	0.479	0.621	0.012
1–1.5	Group	1, 40	9.895	**0.003**	0.198
	Block	2, 80	54.993	** < 0.001**	0.579
	Group × Block	2, 80	4.771	**0.011**	0.107
1.5–2	Group	1, 40	6.499	**0.015**	0.140
	Block	2, 80	34.205	** < 0.001**	0.461
	Group × Block	2, 80	7.322	**0.001**	0.155
2–2.5	Group	1, 40	4.94	**0.032**	0.110
	Block	2, 80	26.576	** < 0.001**	0.399
	Group × Block	2, 80	13.886	** < 0.001**	0.258
2.5–3	Group	1, 40	4.101	**0.050**	0.093
	Block	2, 80	30.788	** < 0.001**	0.435
	Group × Block	2, 80	14.306	** < 0.001**	0.263

*The models include the effect of block (within-subject factor: VH1–VH3), group (between-subject factor: high versus low fear of heights), and their interaction. Significant effects are bolded.*

### Association Between the Measures of Arousal and Postural Measures

Finally, the VR height exposure-induced changes in the measures of distress and ANS activation and the postural measures were assessed using robust (percentage-bend) correlations in the whole sample. The analysis (see Table 5) indicated that the increase in PD (ΔPD) was positively associated with the changes in velocity of CoP deviations (ΔV_*AP*_ and ΔV_*ML*_) as well as the power of CoP displacement at higher frequency ranges (i.e., ΔPSD from 1 to 3 Hz), whereas PD negatively correlated with the power of low-frequency oscillations (ΔPSD 0–0.5 Hz). Similar pattern of association, but weaker correlation, was observed between postural measures and the increase in HR (ΔHR) and SCL (ΔSCL). The decrease in TEMP (ΔTEMP) in response to height exposure was not reliably associated with postural changes.

**TABLE 5:** Correlation coefficients (robust percentage-bend correlation analysis) between the changes in psychophysiological and postural measures induced by exposure to virtual height.

	Δ PD	Δ SCL	Δ HR	Δ TEMP
ΔV_*AP*_	**0.418** **	0.205	0.261^†^	0.057
ΔRMS_*AP*_	0.130	–0.107	0.034	–0.033
ΔV_*ML*_	**0.485** **	0.219	**0.382** *	0.014
ΔRMS_*ML*_	0.175	0.059	0.253	–0.011
ΔPSD 0–0.5 Hz	−0.321*	–0.192	–0.134	–0.184
ΔPSD 0.5–1 Hz	–0.086	0.149	–0.124	0.042
ΔPSD 1–1.5 Hz	**0.489** **	**0.386** *	**0.398** *	–0.033
ΔPSD 1.5–2 Hz	**0.470** **	**0.414** **	**0.341** *	0.269^†^
ΔPSD 2–2.5 Hz	**0.581** ***	0.286^†^	**0.484** **	0.115
ΔPSD 2.5–3 Hz	**0.618** ***	**0.431** **	**0.436** **	0.128

The significance levels for the correlations are marked as follows: ^†^p < 0.10, *p < 0.05, **p < 0.01, ***p < 0.001 (two-tailed, uncorrected). Significant effects are bolded.

*PD, perceived distress; SCL, skin conductance level; HR, heart rate; TEMP, peripheral skin temperature; V_AP_, velocity of CoP displacement in anterior–posterior direction; V_ML_, velocity of CoP displacement in medio-lateral direction; RMS_AP_, root mean square CoP deviation in anterior–posterior direction; RMS_ML_, root mean square CoP deviation in medio-lateral direction; PSD, mean power spectral density of CoP displacement in anterior–posterior direction.*

## Discussion

Our results show that exposure to height in VR reliably evokes a realistic experience accompanied by psychological distress, physiological stress response and changes in postural stability, which are all enhanced in individuals who suffer from fear of heights. We revealed a strong association between the measures of balance control and the markers of distress and autonomic arousal, confirming that postural adaptations are an integral part of the protective reaction to the threat of height.

### Psychophysiological Measures

The efficacy of height exposure using VR is reflected by the extent to which participants feel immersed and present in the virtual scenery. All participants in our study confirmed they had experienced realistic immersion in the VR environments with high level of involvement and interface quality. Exposure to virtual height evoked a significant distress and sympathetic arousal, which were substantially stronger in participants afraid of height. It is probable that standing on the soft surface, which affects the proprioceptive information and amplifies the destabilising effect of height (Simeonov et al., 2005), increased the insecurity and the stress response. This could also contribute to anticipatory distress and arousal in high fear participants at the ground level (stage *VH1*) since no group differences were found at baseline or during recovery stage. Another aspect that could contribute to a reliable psychophysiological activation was the specific type of simulation we used, i.e., a continuous height increase of an open-air elevator. Such a VR environment might be more salient and efficient than, e.g., a sudden exposure to a random virtual height (as used by Wuehr et al., 2019, who did not find any significant increase in HR and EDA). Taken together, our results show that a naturalistic open-air VR simulation of standing at height combined with a destabilising support surface evokes a reliable distress and activation of the sympathetic ANS, which are, as expected, stronger in individuals with fear of heights. Our findings from VR are thus in line with those from the studies of real heights, which have shown that height exposure evokes a significant stress response, in particular in individuals afraid of heights (Meehan et al., 2005; Simeonov et al., 2005; Brown et al., 2006; Davis et al., 2009; Huffman et al., 2009; Cleworth et al., 2012; Zaback et al., 2015, 2019, 2021; Peterson et al., 2018; Martens et al., 2019), indicating a good face, construct and ecological validity of our VR setup and its suitability for future applications.

### Postural Measures and Association With Psychophysiological Measures

With ascending virtual height, high fear participants showed an increase in the velocity of body sway, which was not seen in low fear individuals. This result is similar to that by Wuehr et al. (2019) who reported a positive association between body sway velocity and trait visual height intolerance. Velocity of CoP displacements is sensitive to postural control strategy: slower CoP deviations indicate the engagement of feedback-based control whereas faster sway suggests that feedback is less utilised (Robert et al., 2016). Thus, the increased body sway velocity in high fear participants suggests that they may not be able to use sensory feedback adequately when exposed to heights. We also observed that the magnitude of body sway decreased with ascending virtual height (similarly in both groups), which is in line with some of the previous reports (Cleworth et al., 2012; Raffegeau et al., 2020), but not others (Simeonov et al., 2005; Wuehr et al., 2019; Chander et al., 2021). The reasons for such inconsistent findings across the studies are not clear. For instance, the simulated altitude varies largely across the experiments (as much as by a factor of 10), but there seems to be no clear relationship between the actual elevation and the reported body sway magnitude. Adkin and Carpenter (2018) argued that smaller amplitude of CoP displacements might be related to height-induced threat, while larger deviations are more commonly observed in association with the threat of perturbation. Our results fit into this view, but more empirical evidence is needed to test this hypothesis more directly.

Furthermore, we performed power spectrum analysis of body sway to characterise in more detail the relation between postural adaptations, fear of heights and arousal during simulated height exposure. These analyses have not been carried out in previous VR studies, as far as we know. It has been shown that low frequencies of body sway are associated with visual regulation, medium frequencies with vestibular and somatosensory regulation, and high frequencies with proprioceptive regulation (Nagy et al., 2004). Gradual elevation of VR height decreased low-frequency (<0.5 Hz) and increased high-frequency (>1 Hz) oscillations of body sway, which is similar to real height exposure (Zaback et al., 2019, 2021). However, these changes were more pronounced in the group with high fear compared to low fear individuals. Research has shown that individuals with fear of heights are more dependent on visual information to control balance (see review by Teggi et al., 2019). Height eliminates the availability of visual cues that can be used for posture control so that the reliance on proprioceptive information increases to maintain proper balance (Bles et al., 1980; Salassa and Zapala, 2009). In individuals with fear of height, the visual destabilisation due to simulated height exposure thus seems to have a stronger impact. Consequently, this leads to abnormally elevated postural stiffening, a protective reaction that prevents posture destabilisation and is characterised by increased velocity of CoP displacement and high-frequency body sway (Adkin et al., 2000; Brown et al., 2006; for review see Adkin and Carpenter, 2018).

Our analysis further shows that the postural adjustments during height exposure are significantly related to psychological distress and sympathetic arousal. In particular, body sway velocity and the high-frequency CoP oscillations are significantly positively related to PD as well as HR and EDA. On the other hand, the decrease in low-frequency postural oscillations was less strongly correlated with distress and autonomic arousal. This is in agreement with the experiments by Zaback and co-workers using real height exposure (although with much lower elevations compared with our simulations), who showed that the psychophysiological responses are more strongly related to high-frequency than low-frequency CoP oscillations (Zaback et al., 2019, 2021). Our results suggest that the protective postural adjustments when exposed to height (i.e., stiffening) are an inherent part of a complex protective psychological and bodily reaction to the threat of height. Since individuals with fear of height rely relatively more strongly on visual cues for posture control, the decreased availability of such cues at height may result in an exaggerated estimation of the intensity of the danger. Future studies are needed to directly test this possibility since it has potentially important consequences for the therapy of acrophobia using VR (Lindner, 2021). It has been demonstrated that therapeutic interventions against fear of height affect visual perceptual bias (Dreyer-Oren et al., 2019). Training procedures targetting the efficiency of the use of sensory cues for posture control could thus be potentially useful for the reduction of acrophobia symptoms.

### Limitations

First limitation of the current study is that only young healthy subjects were included. Further research should evaluate balance control also in older adults with fear of falling or individuals with balance disorders (e.g., Parkinson’s disease) or high trait anxiety in order to provide further insight to understand the relationship between fear, balance control and sensory reweighting. Furthermore, our measurements during virtual height exposure did not include standing on solid support surface so that next studies may focus on a direct comparison of solid versus soft ground condition on height VR exposure in individuals with fear of heights. Finally, future research is needed to assess the effect of repeated VR height exposure on postural adaptation (see, e.g., Zaback et al., 2019, 2021; for the effects of repeated exposure to real height) to better understand the associations between changes in fear of heights over time and postural responses, which may yield further improvements of the VR training procedures.

## Conclusion

Our findings show that exposure to height in a naturalistic VR environment elicits a complex reaction to threat involving correlated changes of the emotional state, autonomic activity and postural balance, which are exaggerated in individuals with fear of heights. The analysis of postural measures indicates that individuals with high fear of heights react to heights with maladaptive rigidity of posture due to increased weight of visual input for balance control, while the visual information is less reliable at heights. These findings indicate that interventions targetting posture control might help to treat fear of heights, which remains to be explored in future studies.

## Data Availability Statement

The raw data supporting the conclusions of this article will be made available by the authors, without undue reservation.

## Ethics Statement

The studies involving human participants were reviewed and approved by the Local Ethics Committee, Centre of Experimental Medicine, Slovak Academy of Sciences. The patients/participants provided their written informed consent to participate in this study.

## Author Contributions

DB, MM, ZH, FH, and IR contributed to the concept and design of the study. DB, MM, ZH, and JK conducted the data collection and analyses. MM conducted the statistical analyses. DB wrote the first draft of the manuscript. MM, ZH, JK, FH, and IR contributed to review and critique. All authors contributed to the preparation of this manuscript, reviewed the results, and approved the final version of this manuscript.

## Conflict of Interest

The authors declare that the research was conducted in the absence of any commercial or financial relationships that could be construed as a potential conflict of interest.

## Publisher’s Note

All claims expressed in this article are solely those of the authors and do not necessarily represent those of their affiliated organizations, or those of the publisher, the editors and the reviewers. Any product that may be evaluated in this article, or claim that may be made by its manufacturer, is not guaranteed or endorsed by the publisher.
