# Supplementary material for: The Effects of Virtual Height Exposure on Postural Control and Psychophysiological Stress Are Moderated by Individual Height Intolerance
Source: Front Hum Neurosci. 2022 Jan 12;15:773091. doi: 10.3389/fnhum.2021.773091 (PMC8789875; doi:10.3389/fnhum.2021.773091)
Supplement: Supplementary file 1 [file Data_Sheet_1.docx]

**The effects of virtual height exposure on postural control and psychophysiological stress are moderated by individual height intolerance**

**Supplementary Information**

Diana Bzdúšková, Martin Marko, Zuzana Hirjaková, Jana Kimijanová, František Hlavačka, Igor Riečanský

# Supplementary Methods

**The questionnaire assessing the technical quality of the simulation and the virtual reality experience**

1. **Questions on technical quality:**

Was wearing the VR device comfortable?

Was the VR environment displayed smoothly?

Was the visual and sound design of a good quality?

Was the simulation responding to your movement (e.g., turning your head, tilting) accurately?

1. **Questions on the VR experience:**

To what extent did the virtual environment appear natural to you?

How emotionally engaging was the experience in virtual reality?

How deeply did you immerse in the VR experience?

To what extent did the emotions and feelings you experienced were real?

How strongly did you feel connected to the virtual environment?

How much did the virtual simulation seem authentic to you?

To what extent did the virtual environment mimic the real-world situation?

To what extent was your experience disturbed by external distractors?

**Response scale:**

[0] Not at all, [1] Somewhat, [2] Moderately, [3] Much, [4] Very much.

***
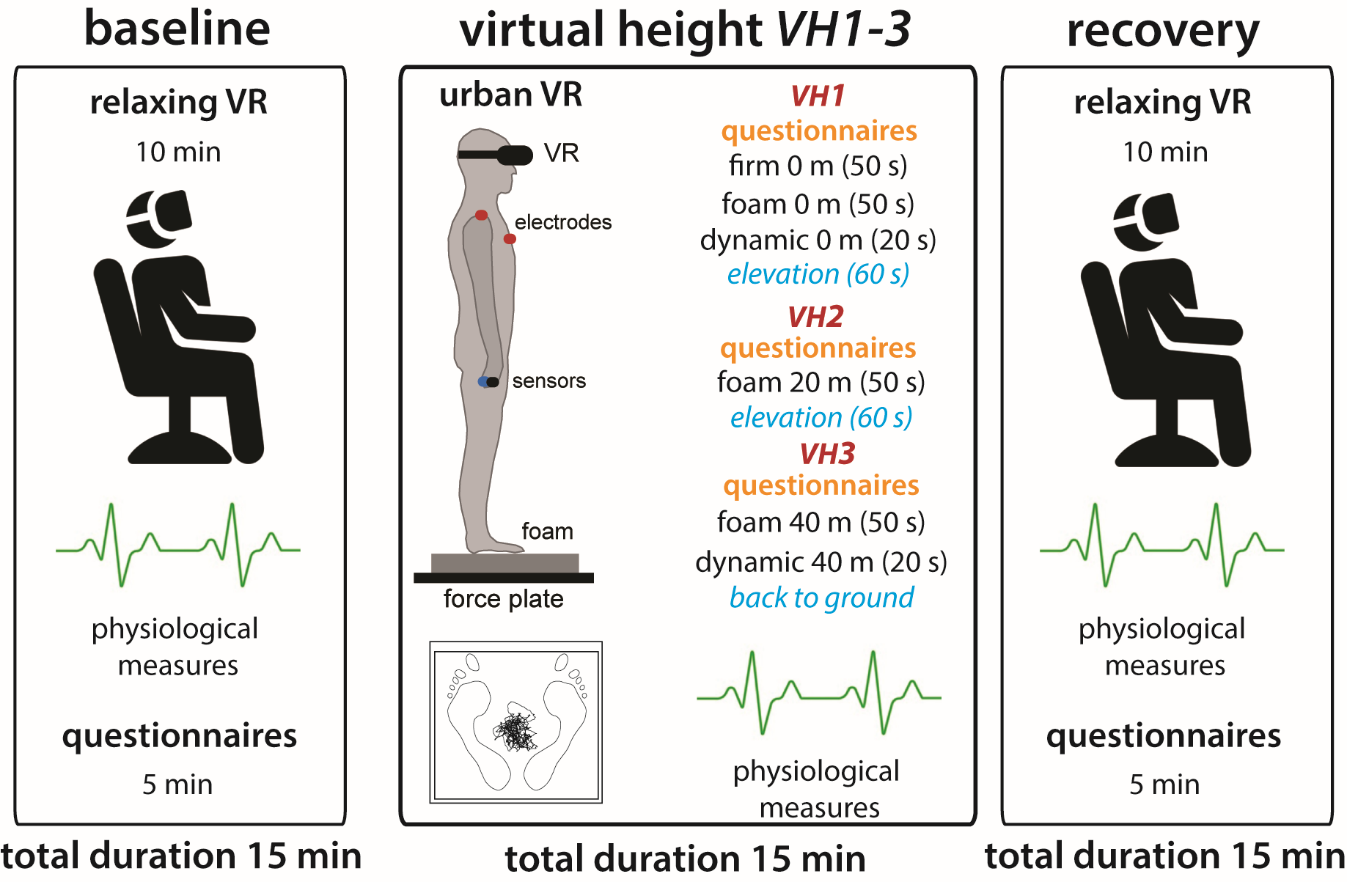
***

***Supplementary Figure 1.*** Schematic representation of the complex VR procedure.

# Supplementary Results

***Supplementary Table.*** Summary of linear mixed effect models LMEMs for postural measures on firm and foam surface. The models included the effect of *surface* (within-subject factor: firm and foam support on ground level – virtual height 0 m), *group* (between-subject factor: high versus low fear of heights) and their interaction. V_AP_ – velocity of CoP in anterior-posterior direction; V_ML_ – velocity of CoP in medio-lateral direction; RMS_AP_ – root mean square of CoP in anterior-posterior direction; and RMS_ML_ – root mean square of CoP in medio-lateral direction. Significant effects are bolded.

| **Measure** | **Effect** | ***df*** | ***F*** | ***p*** | ***R^2^*** |
| --- | --- | --- | --- | --- | --- |
| V_AP_ | Group | 1, 40 | 0.235 | 0 .630 | 0.006 |
|  | Surface | 1, 40 | 719.905 | **< .001** | 0.947 |
|  | Group × Surface | 1, 40 | 0.063 | 0.804 | 0.002 |
|  |  |  |  |  |  |
| V_ML_ | Group | 1, 40 | 1.139 | 0.292 | 0.028 |
|  | Surface | 1, 40 | 252.516 | **< .001** | 0.863 |
|  | Group × Surface | 1, 40 | 5.766 | **0.021** | 0.126 |
|  |  |  |  |  |  |
| RMS_AP_ | Group | 1, 40 | 0.959 | 0 .333 | 0.023 |
|  | Surface | 1, 40 | 330.842 | **< .001** | 0.892 |
|  | Group × Surface | 1, 40 | 4.041 | 0 .051 | 0.092 |
|  |  |  |  |  |  |
| RMS_ML_ | Group | 1, 40 | 1.191 | 0.282 | 0.029 |
|  | Surface | 1, 40 | 268.951 | **< .001** | 0.871 |
|  | Group × Surface | 1, 40 | 6.628 | **0.014** | 0.142 |


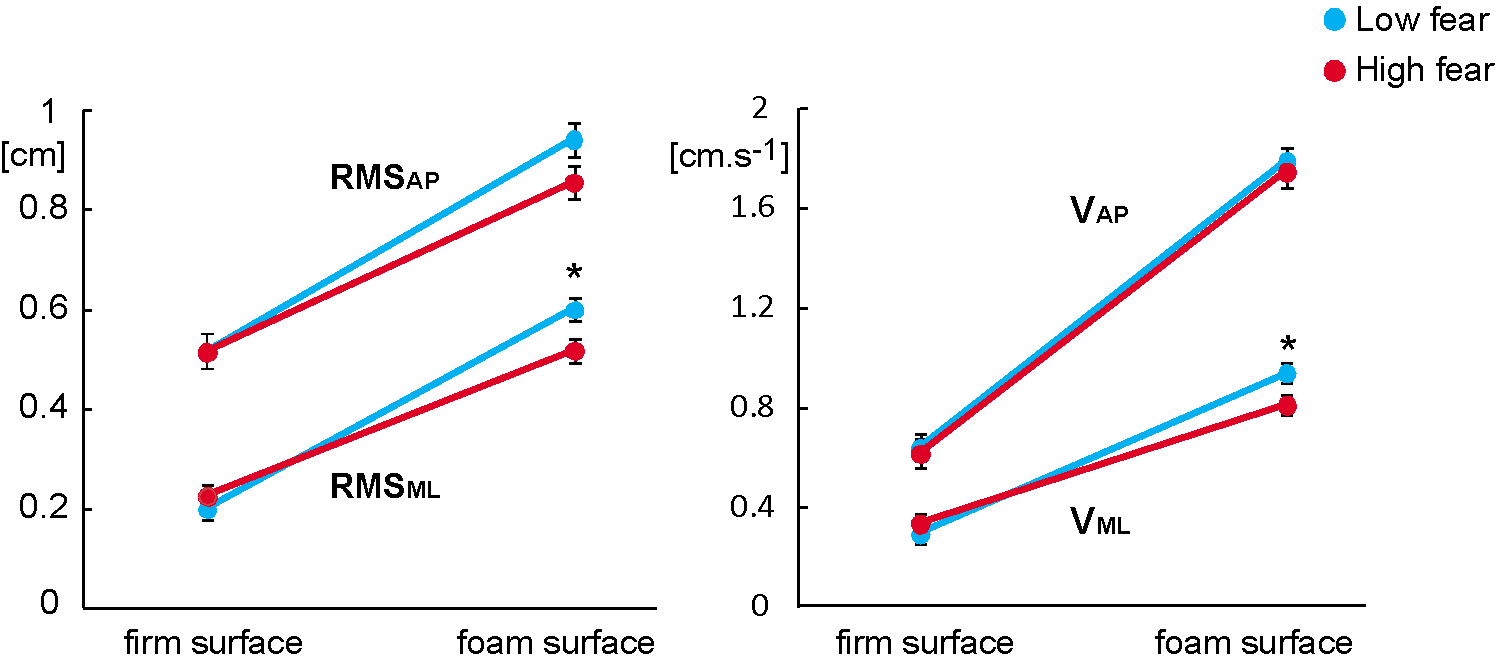


***Supplementary Figure 2.*** Estimated marginal means ± SEM of the RMS and CoP velocity in both directions during stance on firm and foam support surface in ground level in low and high fear groups. Significant effects are bolded. V_AP_ – velocity of CoP in anterior-posterior direction; V_ML_ – velocity of CoP in medio-lateral direction; RMS_AP_ – root mean square of CoP in anterior-posterior direction; and RMS_ML_ – root mean square of CoP in medio-lateral direction.

**
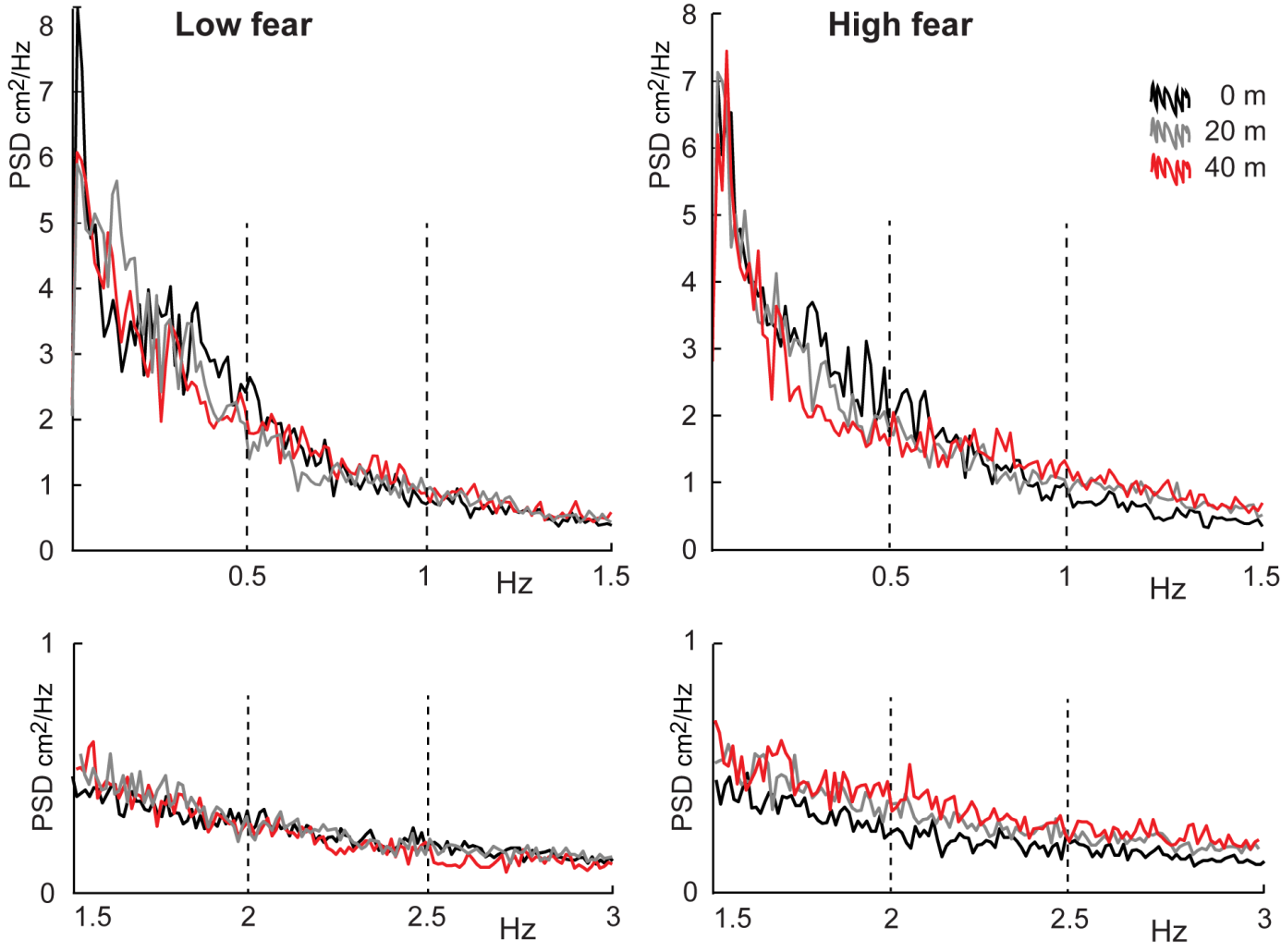
**

***Supplementary Figure 3.*** Mean CoP power spectral density (PSD) in the anterior-posterior direction in virtual heights 0 m, 20 m and 40 m.
